# Chronic Disease Management of Early Childhood Dental Caries: Practices of US Pediatric Dentists

**DOI:** 10.5888/pcd22.240151

**Published:** 2025-01-02

**Authors:** Burton L. Edelstein, Charles E. Basch, Patricia Zybert, Randi L. Wolf, Christie L. Custodio-Lumsden, June Levine, Raynika Trent, Ivette Estrada, Pamela A. Koch, Howard F. Andrews, Carol Kunzel

**Affiliations:** 1 Columbia University Irving Medical Center, New York, New York; 2 Teachers College, Columbia University, New York, New York; 3 Division of Foundational Sciences, Columbia University College of Dental Medicine, New York, New York; 4 Section of Growth and Development, Columbia University College of Dental Medicine, New York, New York

## Abstract

**Introduction:**

Early childhood caries (ECC), dental cavities in children younger than 6 years, is common, consequential, and inequitably concentrated among socially disadvantaged children. The World Health Organization and authoritative clinical and public health agencies promote 4 chronic disease management (CDM) approaches that are low-cost and can be delivered in home and community sites: pharmacologic, behavioral, monitoring, and minimally invasive dentistry (MID). The extent of adoption of these approaches among US pediatric dentists is unknown.

**Methods:**

From November 2021 through July 2023, trained research staff members administered and videorecorded via Zoom a semistructured survey on ECC management to 1,639 clinically active pediatric dentists in the US, including 170 thought leaders (organizational and academic leaders). Data collected included treatment approaches, time allocated to counseling, and personal, practice, and patient population characteristics.

**Results:**

The survey response rate was 27.7%. Among CDM approaches, 88.7% cited pharmacologic approaches, 43.4% behavioral, 41.1% monitoring, and 39.3% MID approaches. MID was significantly associated with thought leaders and with more recent graduates engaged as associates in larger practices or in safety-net settings serving high volumes of low-income children and children with a history of caries. We noted fewer significant associations between other CDM approaches and the characteristics of dentists, practices, and populations served. CDM was not associated with the race or ethnicity of dentists or patients, the numbers of ancillary personnel in practice, or dental management organizations. One-third (32.4%) of respondents reported scheduling 5 or fewer minutes for counseling on caries.

**Conclusion:**

Except for pharmacologic treatments and despite professional guidelines, CDM approaches are underused. We posit that CDM approaches hold strong promise to enhance oral health equity as value-based care arrangements expand in dentistry.

**Summary**

**What is already known on this topic?**

Early childhood caries (ECC) is common, inequitably distributed, consequential, and chronic. Professional guidelines promote chronic disease management (CDM), including pharmacologic, behavioral, monitoring, and minimally invasive dentistry (MID) treatments that reduce the need for costly repair. Unknown is US pediatric dentists’ adoption of CDM for ECC.

**What is added by this report?**

This report of US pediatric dentists’ management of ECC found widespread adoption of pharmacologic treatments but relatively little adoption of behavioral, monitoring, and MID treatments.

**What are the implications for public health practice?**

CDM interventions for ECC are low cost and can be delivered outside of dental facilities by various lay and professional providers to reduce ECC inequities and disease burden.

## Introduction

Despite reductions in dental caries among US children, this chronic disease persists as a clinical and public health challenge that is inequitably concentrated in socially disadvantaged children (1). Nearly 1 in 4 (23%) children aged 2 to 5 years have had primary tooth caries, and 10% of these children have untreated caries (2). Young Black and Hispanic children and children in low-income families have twice the rate of untreated caries as their peers. Untreated, early childhood caries (ECC) — dental cavities (cavitated carious lesions) in children younger than 6 years — results in pain, infection, dysfunction, impaired growth, and reduced quality of life (3), yet it is preventable and suppressible through dietary, hygienic, and fluoride-use behaviors (4). The World Health Organization (WHO) (5) and medical (6), dental (7,8), and public health organizations (9) promote focused early intervention, counseling, fluorides, and early dental care. Many organizations frame guidelines in a health equity lens, noting that ECC reflects social and economic disadvantage.

The American Academy of Pediatric Dentistry (AAPD) (8) and the Association of State and Territorial Dental Directors (ASTDD) (9), from respective clinical and public health perspectives, promote chronic disease management (CDM) strategies, including pharmacologic, behavioral, monitoring, and minimally invasive dentistry (MID) treatments. Community-based early childhood programs, including Head Start and the Special Supplemental Nutrition Program for Women, Infants, and Children (WIC), have adopted individualized risk-based caries management, motivational interviewing, and providers who are not traditional in dentistry (dental therapists, community health workers, dietitians, and health educators) to educate, guide, and support families’ adoption of healthy behaviors (10). CDM research trials evince successful caries suppression (11,12).

Unknown is how extensively pediatric dentists have adopted CDM strategies. This study of US pediatric dentists describes their adoption of CDM to manage ECC. We hypothesized significant variability in CDM adoption and in the amount of time dentists allocate to behavioral counseling based on their personal characteristics and the characteristics of their practice and population served.

## Methods

Eligible for this cross-sectional observational study were all US pediatric dentists who are active members of AAPD, which reports that its membership comprises 87% of all pediatric dentists (Suzanne Wester, Vice President for Membership and Chapter Relations, AAPD, email, July 1, 2024). Our sampling frame was AAPD’s 2021 membership database, which included names, email addresses, zip codes, birth year, and gender for 5,926 active members. The database included 170 dentists that we designated as thought leaders based on 1 or more of their roles as national officers (n = 17), state presidents (n = 45), academic pediatric dentistry chairs (n = 40), and postdoctoral program directors (n = 82). We invited dentists in random order to participate in 45-minute interviews via Zoom.

From November 1, 2021, through July 30, 2023, we mailed personalized prenotification letters on AAPD letterhead to pediatric dentists. The letter was cosigned by the principal investigator (C.K.) and the president, the chief executive officer, and the chief policy officer of AAPD. The letter introduced the study, stated AAPD’s endorsement, encouraged participation, detailed the remote-recorded Zoom format, and offered compensation of a $150 bankcard upon interview completion. One week later, we emailed study details, including expected time commitment and how to schedule a session on Zoom (Zoom Video Communications) using a calendaring application (Calendly). The letter highlighted the value of their input for refining AAPD’s caries management policies. For nonrespondents, we emailed 4 weekly reminders, 3 more weekly reminders 3 months after initial contact, and 1 final reminder as the study closed.

Following a scripted interview guide that included closed- and open-ended questions, our 15 trained and monitored interviewers conducted and recorded interviews to obtain information on participants’ CDM management practices, including time devoted to behavioral counseling and personal, practice, and population characteristics. 

For personal characteristics, interviewers asked participants at the close of the interview to self-report their age, gender, race, ethnicity, year of dental school graduation, and practice role. For practice characteristics, they also asked for zip code, practice type, practice size, and affiliation with dental management or dental support organizations. At the beginning of each interview respondents were asked to estimate the percentage of children in their practice who are aged 6 years or younger, have any early childhood caries experience, and have rampant or severe early childhood caries experience as defined by AAPD. At the end of the interview, respondents were asked to estimate the percentage of children by type of insurance coverage (private/commercial, public, military, Indian Health Service, other, and none), by income (“poor or low-income/working poor” defined for the respondent as being from a US mainland family with an “annual family income for a family of 4 under $52,400” [or other income level for Alaska and Hawaii] based on the federal Department of Health and Human Services Poverty Guidelines), and by race and by ethnicity. 

For analyses, we created categories for the following characteristics: age (<40, 41–50, 51–60, and >60 y); gender (male or female; no respondent stated any other descriptor); race (Asian, Black, White, or “Other,” which included Hawaiian/Other Pacific Islander, Native American/Alaska Native, and multiple or other); ethnicity (Hispanic or non-Hispanic); year of graduation from dental school (1970–1979, 1980–1989, 1990–1999, 2000–2009, or 2010 and thereafter); and practice role (owner or associate). We categorized practice characteristics for location by using zip code to assign location to one of 9 regions established by the US Census Bureau (New England, Middle Atlantic, East North Central, West North Central, South Atlantic, East South Central, West South Central, Mountain, and Pacific); for practice type (private practice or not in private practice); for practice size as number of staff members (dentists, hygienists, assistants, dental therapists, and other [including clerical, care coordinators, health educators, outreach workers, community health workers, dieticians, social workers] as 0, 1, 2, 3, 4–6, 7–10, and ≥11); and for affiliation with management and support organizations as yes or no. We categorized estimated percentage of children in the practice by insurance type, percentage of children who are from poor or low-income/working-poor families, race and ethnicity, percentage of children aged under 6 years, percentage aged under 6 years with caries experience, and percentage of children aged under 6 years with rampant or severe caries experience as 0% to 25%, 26% to 50%, 51% to 75%, and more than 75%.

Video and audio recordings were retained and autotranscribed by Zoom for later review and analysis. Interviewer training involved 15 hours of preparation using 11 learning modules covering human subjects research, interviewing techniques, and observed mock interviews. We monitored interviewers’ performance by reviewing 2 interviews each month by each interviewer.

We asked the following question without probing or specifying the 4 CDM approaches: “For pre-cooperative children — that is, children who are developmentally too young to tolerate conventional restorative treatment — how do you and your staff currently manage ECC?” Interviewers, using predetermined lists of treatments in each category, coded responses into as many CDM treatment categories — pharmacologic, behavioral, monitoring, and MID — as the respondent mentioned. If unsure, interviewers coded a response as “other” and the research team’s pediatric dentist (B.L.E.) assigned such responses to 1 of the 4 CDM categories. We coded any reference to topical agents (eg, silver diamine fluoride, fluoride varnish, iodine) as pharmacologic and any approaches that did not involve intraoral manipulation as behavioral, including counseling, advising, instructing, directing, and motivational interviewing. The category monitoring captured responses such as increased frequency of dental visits, watchful waiting, and active surveillance. MID included atraumatic restorative technique (ART), silver-modified ART, interim therapeutic restorations, Hall crowns, glass ionomer, sealants, resin infiltration, and excavation without restoration.

Study data were securely collected by using REDCap (Research Electronic Data Capture) (13,14) and stored and managed in New York State Psychiatric Institute’s Data Coordinating Center. To protect against data disclosure and loss of confidentiality, we encrypted and double-password protected study computers. We stored recordings and transcriptions on a credential-protected secure Google shared drive and a digital media video storage platform. We used SPSS Statistics version 29.0 (IBM Corporation) for data analysis. Any finding with a 2-sided *P* <.05 was considered to be significant. We calculated descriptive statistics for the characteristics of respondents and their practices and patient populations served. We used χ^2^ tests to assess differences in rates of reported treatment modalities and applied continuity corrections to bring normal curve probabilities into closer agreement with binomial probabilities (ie, in 2 × 2 tables).

The Columbia University Medical Center’s and Teachers College’s institutional review boards (AAAT4950, 22029) approved this study.

## Results

Of the 5,926 AAPD members approached, 1,639 completed the interview, yielding an overall response rate of 27.7%. The rate was higher among thought leaders than among other pediatric dentists (52.9% vs 26.9%, χ^2^ with continuity correction = 54.6, *P* < .001) and higher among younger dentists: 34.9% among respondents younger than 40 years, 24.3% among those aged 41 to 50 years, 24.1% among those aged 51 to 60 years, and 23.8% among those older than 60 years (χ^2^ for linear trend = 47.5, *P* < .001). The response was also higher among females than males (29.9% vs 24.4%, χ^2^ with continuity correction = 21.1, *P* < .001). Gender and age were strongly associated: female dentists comprised 68.9% of respondents younger than 40 years, 58.4% aged 41 to 50 years, 52.5% aged 51 to 60 years, and 41.4% older than 60 years (χ^2^ for linear trend = 168.2, *P* < .001). Response rates of dentists differed by geographic location: 35.0% on military bases, 33.6% in Mid East states, 30.5% in Far West, 28.7% in New England, 28.1% in Rocky Mountain, 27.2% in Great Lakes, 26.7% in Plains, 25.8% in Southwest, and 21.2% in Southeast (χ^2^ = 54.0, *P* < .001), but we found no differences in rates between the 10 largest metropolitan areas and other areas.

The most common reasons for nonresponse were failure to reply (n = 3,898; 90.9% of 4,288 nonresponders); failure to keep an appointment (n = 223; 5.2%); email delivery failure (n = 110, 2.6%), ineligibility (n = 41, 1.0%), and lack of availability or interest (n = 15, 0.3%).

The sample self-reported as predominantly White (66.5%) and non-Hispanic (93.6%) (Table 1). Thought leaders were older than other pediatric dentists (16.7% vs 45.1% were aged ≤40 y; 26.7% vs 6.4% were aged >60 y), graduated from dental school earlier (11.1% vs 0.3% graduating before 1980; 12.2% vs 45.5% graduating in 2010 or later), and were less often in private practice (44.9% vs 90.1%). The percentage of dentists that served the lowest-income patient population (>75% of patient population is poor or low-income) was higher among thought leaders than among other pediatric dentists (39.5% vs 12.6%). Similarly, the percentage of dentists that served the highest-income patient population (<25% of patient population is poor or low-income) was higher among other pediatric dentists than among thought leaders (44.8% vs 18.6%).

**Table 1:** Characteristics of Respondents (N = 1,639) to an Interview Survey of US Pediatric Dentists Conducted by Columbia University, November 2021–July 2023^a^

Characteristic	No. (%)^b^			χ^2^ test statistic (*P* value)^d^
	All (N = 1,639)	Thought leaders^c^ (n = 90)	Other pediatric dentists (n = 1,549)	
**Age, y**				
≤40	713 (43.5)	15 (16.7)	698 (45.1)	54.9 (<.001)
41–50	539 (32.9)	31 (34.4)	508 (32.8)	
51–60	264 (16.1)	20 (22.2)	244 (15.8)	
>60	123 (7.5)	24 (26.7)	99 (6.4)	
**Gender**				
Male	1,046 (63.8)	50 (55.6)	996 (64.3)	2.5 (.12)
Female	593 (36.2)	40 (44.4)	553 (35.7)	
**Race**				
Asian	406 (25.5)	21 (23.3)	385 (25.6)	0.30 (.86)
White	1,058 (66.5)	61 (67.8)	997 (66.4)	
Other^e^	127 (8.0)	8 (8.9)	119 (7.9)	
**Ethnicity**				
Non-Hispanic	1,525 (93.6)	84 (93.3)	1,441 (93.6)	0 (>.99)
Hispanic	105 (6.4)	6 (6.7)	99 (6.4)	
**Year of dental school graduation**				
1970–1979	14 (0.9)	10 (11.1)	4 (0.3)	90.8 (<.001)
1980–1989	122 (7.5)	17 (18.9)	105 (6.8)	
1990–1999	239 (14.6)	22 (24.4)	217 (14.0)	
2000–2009	547 (33.4)	30 (33.3)	517 (33.4)	
2010 or later	715 (43.7)	11 (12.2)	704 (45.5)	
**Practice type**				
In private practice	1,431 (87.6)	40 (44.9)	1,391 (90.1)	154.1 (<.001)
Not in private practice	202 (12.4)	49 (55.1)	153 (9.9)	
**% of Patient population that is poor or low income**				
0–25	673 (43.4)	16 (18.6)	657 (44.8)	53.5 (<.001)
26–50	420 (27.1)	17 (19.8)	403 (27.5)	
51–75	239 (15.4)	19 (22.1)	220 (15.0)	
>75	219 (14.1)	34 (39.5)	185 (12.6)	

a Interviews were conducted via Zoom.

b Ns vary slightly between variables due to sporadic missing data; percentages may not add to 100 because of rounding.

c Thought leaders were state and national officers of the American Academy of Pediatric Dentistry and dental educators.

d χ^2^ with continuity correction for gender and ethnicity. χ^2^ for linear trend for age, year of dental school graduation, and % poor or low income. *P* <.05 considered significant.

e Includes Black, Native American/Alaska Native, Hawaiian/Other Pacific Islander, and multiple races.

Among all respondents, 94.1% reported using 1 or more CDM approaches: pharmacologic (88.7%), behavioral (43.4%), monitoring (41.1%), and MID (39.3%) (Table 2). Among 1,454 dentists reporting pharmacologic approaches, the most frequently cited were silver diamine fluoride (n = 1,381; 95.0%) and fluoride varnish (n = 585; 40.2%). Among the 644 reporting MID approaches, the most frequently cited were ART or interim therapeutic restorations (n = 299; 46.4%), Hall crowns (n = 255; 39.6%), and glass ionomer cements (n = 177; 27.5%).

**Table 2:** Chronic Disease Management Approaches to Managing Early Childhood Caries Among Respondents (N = 1,639) to an Interview Survey of US Pediatric Dentists Conducted by Columbia University, November 2021–July 2023^a^

Characteristic	No.	Chronic disease management approach							
		Pharmacologic		Behavioral		Monitoring		Minimally invasive dentistry	
		No. (%)^b^	χ^2^ (*P* value)^c^	No. (%)^b^	χ^2^ (*P* value)^c^	No. (%)^b^	χ^2^ (*P* value)^c^	No. (%)^b^	χ^2^ (*P* value)^c^
**All**	1,639	1,454 (88.7)	—	712 (43.4)	—	673 (41.1)	—	644 (39.3)	—
**Type of dentist**									
Thought leaders^d^	90	78 (86.7)	0.2 (.65)	47 (52.2)	2.6 (.10)	27 (30.0)	4.3 (.04)	45 (50.0)	4.1 (.04)
Other pediatric dentists	1,549	1,376 (88.8)		665 (42.9)		646 (41.7)		599 (38.7)	
**Age, y**									
≤40	713	660 (92.6)	31.0 (<.001)	314 (44.0)	0.1 (.76)	325 (45.6)	15.1 (<.001)	313 (43.9)	9.8 (.002)
41–50	539	479 (88.9)		223 (41.4)		217 (40.3)		201 (37.3)	
51–60	264	217 (82.2)		130 (49.2)		93 (35.2)		85 (32.2)	
>60	123	98 (79.7)		45 (36.6)		38 (30.9)		45 (36.6)	
**Year of dental school graduation**									
1970–1979	14	10 (71.4)	36.7 (<.001)	4 (28.6)	0.1 (.82)	3 (21.4)	15.8 (<.001)	7 (50.0)	6.6 (.01)
1980–1989	122	94 (77.0)		48 (39.3)		39 (32.0)		46 (37.7)	
1990–1999	239	200 (83.7)		121 (50.6)		84 (35.1)		74 (31.0)	
2000–2009	547	486 (88.8)		222 (40.6)		220 (40.2)		205 (37.5)	
2010 or after	715	662 (92.6)		316 (44.2)		326 (45.6)		311 (43.5)	
**% of Patient population that is poor or low income**									
0–25	673	596 (88.6)	0.1 (.80)	288 (42.8)	1.0 (.32)	275 (40.9)	0.4 (.51)	238 (35.4)	10.9 (<.001)
26–50	420	377 (89.8)		192 (45.7)		179 (42.6)		173 (41.2)	
51–75	239	207 (86.6)		105(43.9)		93 (38.9)		96 (40.2)	
>75	219	198 (90.4)		82 (37.4)		85 (38.8)		106 (48.4)	

a Interviews were conducted via Zoom.

b Ns vary slightly between variables due to sporadic missing data.

c χ^2^ with continuity correction for type of respondent. χ^2^ for linear trend for age, year of dental school graduation, and % poor or low income. *P* <.05 considered significant.

d Thought leaders were state and national officers of the American Academy of Pediatric Dentistry and dental educators.

Those citing pharmacologic, monitoring, or MID were younger, graduated from dental school more recently, and had fewer years in practice. Differences in rates between youngest and oldest age groups were 12.9 percentage points (92.6% vs 79.7%) for pharmacologic, 7.4 percentage points for behavioral (44.0% vs 36.6%), 14.7 percentage points for monitoring (45.6% vs 30.9%), and 7.3 percentage points for MID (43.9% vs 36.6%). MID was more often used in lower-income and Medicaid-insured patient populations, with a spread from lowest to highest income groups of 13.0 percentage points (35.4% vs 48.4%). Thought leaders had a lower rate than other pediatric dentists of monitoring (30.0% vs 41.7%; χ^2^ = 4.3; *P* = .04) and a higher rate of MID (50.0% vs 38.7%; χ^2^ = 4.1; *P* = .04). We found no differences in rates of behavioral approaches by any study variables other than an association with populations of children younger than 6 years with caries experience: we found a difference of 25.1 percentage points (51.1% vs 26.0%; *P* = .04) between the group with the highest percentage (>75%) and the group with lowest percentage (≤25%) of such experience.

MID was associated with more personal, practice, and population characteristics than other CDM approaches (Table 2). In addition to associations with thought leaders (*P* = .04), younger dentists (*P* = .002), and more recent graduation from dental school (*P* = .01), MID was mentioned more frequently by those having fewer than 6 years of practice than those with more than 30 years of practice (difference of 6.2 percentage points; 44.2% vs 38.0%; χ^2^ = 9.7; *P* *=* .002); by those in practices with 10 or more dentists than solo practitioners (difference of 9.9 percentage points; 47.5% v 37.6%; χ^2^ *=* 5.7; *P* *=* .02); and by dentists not in private practice than those in private practice (difference of 14.5 percentage points; 52.0% vs 37.5%; χ^2^ *=* 8.5; *P* < .001). MID was also cited more often by dentists whose patient population is more than 75% children under age 6 years and children under age 6 with caries experience than those whose patient population is fewer than 26% such children, with spreads of 18.9 and 10.5 percentage points respectively (56.9% vs 38.0%; χ^2^ *=* 8.2; *P* = .004 and 42.3% vs 31.8%; χ^2^ *=*5.0; *P* = .02 respectively), by dentists in practices whose patient population is more than 75% Medicaid-insured children than those whose patient population are less than 26% such children, with a spread of 8.7 percentage points (46.3% vs 37.6%; χ^2^ *=* 4.0; *P* = .05), treat smaller proportions of privately insured patients (≤26%) than publicly insured children (>75%) with a spread of 10.5 percentage points (46.5% vs 36.0%; χ^2^ = 8.5; *P* = .004), and higher proportions of children from poor and low-income families (>75%) than lower proportions (≤26%), with a spread of 13 percentage points (48.4% vs 35.4%; χ^2^ *=* 10.9; *P* < .001).

Not associated with CDM approaches were the race and ethnicity of dentists or patients (except higher rates of pharmacologic approaches among practices serving higher proportions of Asian patients [12.4 percentage point difference between lowest and highest proportion of Asian children; χ^2^ *=* 4.0; *P* = .04]), practice affiliation with dental management organizations, numbers of dental ancillary personnel, and proportion of young children with rampant caries experience.

All but 1 respondent gave a nonzero reply when asked about time allocated to preventive counseling for parents of children with ECC; of 1,633 respondents to this question, 529 (32.4%) reportedly spend 5 minutes or less, 693 (42.4%) from 6 to 10 minutes, and 411 (25.2%) more than 10 minutes (Table 3). Those allocating more time were female (28.9% female vs 38.6% male in the group reporting 0–5 minutes; 27.5% female vs 21.0% male in the group reporting >10 minutes), not in private practice (20.4% not in private practice vs 34.1% in private practice in the group reporting 0–5 minutes; 34.8% not in private practice vs 23.8% in private practice in the group reporting >10 minutes), or thought leaders (24.7% thought leaders vs 32.8% other pediatric dentists in the group reporting 0–5 minutes; 39.3% thought leaders vs 24.4% other pediatric dentists in the group reporting >10 minutes). Age, year of dental school graduation, and proportion of patient population that is poor or low income were not associated with time allocated to preventive counseling.

**Table 3:** Time Allocated to Health Behavioral Counseling for Early Childhood Caries by 1,633 Respondents to an Interview Survey of US Pediatric Dentists Conducted by Columbia University, November 2021–July 2023^a^

Respondent characteristic	No.	Time allocated, no (%) of respondents			χ^2^ (*P* value)^b^
		0–5 min	6–10 min	>10 min	
**All**	1,633	529 (32.4)	693 (42.4)	411 (25.2)	**—**
**Type of dentist**					
Thought leader^c^	89	22 (24.7)	32 (36.0)	35 (39.3)	7.9 (.005)
Other pediatric dentists	1,544	507 (32.8)	661 (42.8)	376 (24.4)	
**Gender**					
Male	590	228 (38.6)	238 (40.3)	124 (21.0)	17.5 (*<*.001)
Female	1,043	301 (28.9)	455 (43.6)	287 (27.5)	
**Practice type**					
In private practice	1,426	486 (34.1)	601 (42.1)	339 (23.8)	19.0 (<.001)
Not in private practice	201	41 (20.4)	90 (44.8)	70 (34.8)	

a Interviews were conducted via Zoom. Not all 1,639 respondents completed all questions.

b χ^2^ with continuity correction for type of respondent. χ^2^ for linear trend for age, year of dental school graduation, and % poor or low income. *P* <.05 considered significant.

c Thought leaders were state and national officers of the American Academy of Pediatric Dentistry and dental educators.

## Discussion

The adoption of a CDM approach for pediatric dental caries is currently promoted in the US and internationally by clinical and public health authorities (4–9,15–20), but its uptake by US pediatric dentists has not been investigated until now. Ours is the largest study to date to examine CDM in pediatric dental practice. Recognizing low response rates to AAPD’s surveys, we used extensive outreach and a large honorarium. After initially rejecting the $150 honorarium as potentially coercive, the IRBs acknowledged its appropriateness in the context of pediatric dentists’ high incomes. The large percentage of respondents who were young and female reflects the growing proportion of female dentists, which increased from 24.1% in 2010 to 34.5% in 2020 (21). Differences in response rate by region raises the possibility of regional differences in CDM adoption, although we found no differences between major metropolitan areas and less populated areas.

Thought leaders, 72% of whom serve as residency program directors or department chairs in academic and hospital institutions that disproportionately provide care to low-income populations (22), used monitoring and MID more than other pediatric dentists but not at rates higher than other pediatric dentists for pharmacologic or behavioral approaches. This finding may reflect the overall high rates of silver diamine fluoride adoption and low rates of behavioral adoption by all pediatric dentists. Thought leaders also reported spending more time on counseling despite providing more care to poor and low-income patients, whose typical health insurance coverage is Medicaid, a less remunerative coverage than private insurance. This finding may reflect an interaction between their commitment to prevention through counseling and presumed lesser perceived demand for income-generating productivity.

The finding that younger dentists, who are also more recent graduates with fewer years of practice, more often cited pharmacologic, monitoring, and MID may reflect advances in dental education that align with professional recommendations. The finding that they, like their older colleagues, infrequently reported using behavioral interventions may reflect a lack of preparation in behavioral counseling and a lack of payment for counseling, particularly dietary counseling (23,24). Unknown are rates of CDM among the 13% of pediatric dentists who are not AAPD members. We conjecture that they may adopt CDM at lower rates than their peers because they may be less familiar with AAPD’s guidelines.

The significant associations between respondent, practice, and population characteristics and MID suggest that this approach is a priority among pediatric dentists, including program directors and academic chairs providing care in safety-net settings that treat large volumes of children from poor and low-income families who are publicly rather than privately insured. Also associated with MID are younger respondents who are generally more recent dental school graduates with fewer years in practice. This finding may reflect both their attraction to providing care in safety-net settings and the numbers of young pediatric dentists who have become program directors in response to the marked increase in programs in recent years.

AAPD promotes CDM through risk assessment and active surveillance of disease progression (16). It advances risk-based care paths that have demonstrated success in controlling ECC with fewer complications and more efficient use of resources (25). ASTDD supplements these principles by “taking care beyond the dental office, improving health outcome tracking, and implementing a family-centered and value-based system of care” (9). Such a system advances holistic, interprofessional team approaches to disease prevention and management. It anticipates a payment environment in which providers are rewarded for outcomes (value) over procedures (volume) to enhance care quality, patient experience, and provider performance (26). WHO endorses each of these strategies and further promotes community water fluoridation, population oral health education, engagement of primary care medical providers, and linking oral health to food policies (5). It notes that prevention is better able to reach populations with the greatest burden of disease and that MID treatments delivered in supportive dental, medical, and community environments can enhance oral health equity.

Because topical fluorides “slow, arrest, or reverse early carious lesions and strengthen developing teeth, making them resistant to future decay” (9), they have become a mainstay of nonrestorative treatment of dental caries (7). Silver diamine fluoride is efficacious, low-cost, easy to apply, and less invasive than restorative care (27). Its adoption is notable as it has been used as an off-label cariostatic agent only since 2016 (28) and is covered by most private insurance (29) and by 38 state Medicaid authorities (30). Among other fluoride preparations, few dentists in our study (4.5%) mentioned fluoridated toothpastes or supplements.

Studies using behavioral approaches, particularly among populations at high risk of ECC, evince the complexity of behavior change even when interventions are grounded in recognized behavioral theories and principles of motivational interviewing (31). AAPD (8), ASTDD (9), and WHO (5) recommend individual and community-level educational interventions based on cariologic and behavioral principles. Improving parental knowledge is necessary but insufficient for adoption of sustained salutary behaviors. But routine counseling on caries prevention, characterized by ASTDD as “uninspired,” (9) has not been demonstrated to be effective (31).

The National Academy of Medicine (formerly the Institute of Medicine) places CDM, including behavioral management, on a continuum of care between prevention and repair, thereby establishing behavioral interventions as a distinct form of treatment (17). Low rates of reporting behavioral interventions may reflect that many dentists seemingly do not regard counseling to be a caries treatment per se, despite all but 1 dentist reporting that they spend some time providing counseling. Modest levels of reporting may also relate to lack of private and public insurance reimbursement. Private dental plans do not typically cover oral health counseling, and few state Medicaid programs pay for caries risk assessment (n = 6, from $0.01 to $15.00 per assessment), nutrition counseling (n = 6 from $10.87 to $38.56 per assessment); no Medicaid programs pay for motivational interviewing (30).

Monitoring is the least specific CDM approach because it has evolved from a concept of passive “watchful waiting” to “active surveillance” in association with other CDM approaches. The finding that thought leaders serving large volumes of low-income patients were less likely than other dentists to employ monitoring (30.0% vs 41.7%) may reflect their older age–associated interpretation of “monitoring” as passive. AAPD’s care paths suggest that the frequency of preventive visits should reflect a child’s individual level of caries risk: low and moderate risk would necessitate semiannual visits and high risk would necessitate quarterly visits (8).

MID arose in the 1990s as a public health approach to treating caries because it can be delivered outside of dental settings (18). WHO has endorsed MID as faster, less expensive, and less technical than conventional dental repair, in addition to being free of mercury, and notes that it is suitable for community settings, including preschools and daycare facilities (19). MID has gained adoption with the advent of adhesive dental materials such as glass ionomer cements and Hall crowns, which are forgiving of technique and can be placed without local or general anesthesia (20,32). Thought leaders more closely followed professional recommendations for emerging MID approaches perhaps because national officers set association policy, while state leaders and academic pediatric dentists lead the profession in implementing policy.

### Limitations

Our study has several potential limitations. The results may not be generalizable to all US pediatric dentists due to the study’s sampling methods. However, this large sample provides a substantive snapshot of CDM adoption among pediatric dentists, offering insights that hold promise for reducing inequities through the use of lower-cost and more accessible caries treatments compared with conventional methods. The study was cross-sectional and conducted during the COVID-19 pandemic. Its cross-sectional design precludes drawing causal inferences. Generalizing to future periods will require study replication. Our open-ended question on how dentists manage ECC reflects only their top-of-mind responses because we did not probe for further exploration of approaches not mentioned. Individual interviewers, although trained by using a standardized procedure, may have influenced participants’ responses. Self-reports of caries management may reflect social desirability among those who are aware of current caries management recommendations. While we found some differences in CDM practices by provider, practice, and population characteristics, we did not evaluate these characteristics as barriers to or facilitators of CDM. Future studies, including qualitative analyses of our open-ended questions and quantitative analyses of dietary recommendations and clinical experiences, may explore causal relationships and seek to understand barriers and facilitators to CDM adoption.

### Conclusion

As a chronic yet preventable lifestyle disease, dental caries relates to modifiable behavioral risk factors that disproportionately affect disadvantaged populations and result in considerable health inequities (33). Amelioration of such conditions requires coordinated strategies across multiple levels (34). Chronic conditions are amenable to team-based care coordination, delivery of services in novel settings, behavioral interventions, and attention to social determinants. Yet root-cause, multidisciplinary, holistic approaches to caries prevention and management are generally outside the realm of conventional dental practice, a cottage industry largely comprising solo and small providers separate from the education, organization, delivery, and payment mechanisms of mainstream medicine. Barriers to adopting CDM include resistance to change, lack of training in dietary and behavioral sciences, lack of dental coverage for behavioral risk-reduction services, and insufficient integration of community-based public health endeavors with clinical dental care. Additional clinician-related barriers are deficits of knowledge and awareness and misbeliefs about disease, strategies, culture, and guidelines; deficits of skills, proficiencies, and clinical competencies; limitations in perceived roles and responsibilities; lack of motivation and prioritization; and frustration (35).

Addressing ECC through CDM holds promise to reduce ECC inequities across populations. CDM of ECC may be delivered by a wide range of providers — including medical and dental personnel, health educators, community health workers, social workers, and nutritionists — in a wide range of health care, community, and — for behavioral interventions — telehealth settings. Conditions presaging greater adoption of CDM that hold promise to reduce disparities in ECC include greater adoption of CDM by younger pediatric dentists, high levels of MID adoption by thought leaders, and pressures from health reform that prioritize value over volume and accountability to population health.
